# 基于CMR的肺动脉径线测量评价肺高血压的临床实用价值

**DOI:** 10.3779/j.issn.1009-3419.2017.02.10

**Published:** 2017-02-20

**Authors:** 帆 杨, 东 李, 振文 杨, 璋 张, 丹 王, 铁链 于

**Affiliations:** 1 300052 天津，天津医科大学总医院放射科 Department of Radiology, Tianjin Medical University General Hospital, Tianjin 300052, China; 2 300052 天津，天津医科大学总医院心内科 Department of Cardiovascular Disease, Tianjin Medical University General Hospital, Tianjin 300052, China

**Keywords:** 肺高血压, 肺动脉, 心血管核磁共振, Pulmonary hypertension, Pulmonary artery, Cardiovascular magnetic resonance

## Abstract

**背景与目的:**

肺高血压（pulmonary hypertension, PH）将导致主肺动脉扩张，主肺动脉（main pulmonary artery, MPA）径线测量可被临床用于评估PH。本研究旨在探讨心血管磁共振（cardiovascular magnetic resonance, CMR）测量MPA各径线参数评价PH的价值。

**方法:**

对经右心导管检查（right heart catheterization, RHC）确诊的83例PH患者及49例健康志愿者CMR图像进行分析，测量和计算主肺动脉横径（main pulmonary artery diameter, D_PA_）、D_PA_与升主动脉横径之比（the ratio of D_PA_ and ascending aorta diameter, D_PA_/D_Ao_）、最大平均直径（max mean diameter, MDmax）、最小平均直径（min mean diameter, MDmin）、横径变化分数（fraction transverse diameter, fTD）、纵径变化分数（fraction longitudinal diameter, fLD）及MPA顺应性。

**结果:**

PH组的D_PA_、D_PA_/D_Ao_、MDmax、MDmin明显增大（*P* < 0.001），fTD、f LD、顺应性显著减低（*P* < 0.001）。对照组fTD小于f LD（*P* < 0.001），但PH组两参数无明显差异（*P*=0.305）。MPA各径线参数均与平均肺动脉压（mean pulmonary arterial pressure, mPAP）显著相关（*P* < 0.05），以D_PA_/D_Ao_的相关性（*r*=0.534, *P* < 0.001）最强。ROC曲线分析表明MDmin > 28.4 mm和MDmax > 32.4 mm预测PH的效能更高[曲线下面积（area under the curve, AUC=0.979, 0.981）]，并且两者的特异性均达100%。

**结论:**

CMR无创性测量MPA径线评价PH具有临床实用价值。

## Abstract (en)

**Background and objective:**

Pulmonary hypertension (PH) often leads to dilatation of main pulmonary artery (MPA). MPA measurements can be used to predict PH. This aim of this study is to investigate power of MPA vessel indices, which are acquired from cardiovascular magnetic resonance, to evaluate PH.

**Methods:**

Cardiovascular-magnetic-resonancedetermined parameters of MPA were acquired and calculated in 83 PH patients, whose diagnosis were confirmed with right heart catheterization and 49 healthy volunteers; these parameters included MPA diameter (D_PA_), ratio of D_PA_ and ascending aorta diameter (D_PA_/D_Ao_), max mean diameter (MDmax), min mean diameter (MDmin), fraction transverse diameter (fTD), fraction longitudinal diameter (fLD), and distensibility.

**Results:**

Compared with control group, D_PA_, D_PA_/D_Ao_, MDmax, and MDmin were significantly higher in patients with PH (*P* < 0.001); fTD, fLD, and distensibility significantly decreased (*P* < 0.001). fTD was lesser than fLD in control group (*P* < 0.001), whereas difference was not observed in PH (*P*=0.305). MPA indices were significantly correlated with mean pulmonary arterial pressure (mPAP) (*P* < 0.05), and best correlation was observed for D_PA_/D_Ao_ (*r*=0.534, *P* < 0.001). By receiver operating characteristic analysis, MDmin > 28.4 mm, and MDmax > 32.4 mm (area under the curve, AUC=0.979, 0.981) showed best performance in predicting PH, yielding highest specificity at 100%.

**Conclusion:**

Noninvasive cardiovascular-magnetic-resonance-derived MPA measurements provide excellent and practical reference in clinical settings for detecting PH.

肺高血压（pulmonary hypertension, PH）被定义为经右心导管（right heart catheterization, RHC）测得静息状态下平均肺动脉压（mean pulmonary arterial pressure, mPAP）≥25 mmHg的血液动力学和病理生理学异常状态^[1]^。计算机体层成像（computed tomography, CT）和心血管磁共振（cardiovascular magnetic resonance, CMR）是目前无创评估PH的重要影像学方法，但却不能直接测量肺循环的压力。CT常以主肺动脉横径（main pulmonary artery diameter, D_PA_）≥29 mm^[2-4]^、D_PA_与升主动脉横径之比（the ratio of D_PA_ and ascending aorta diameter, D_PA_/D_Ao_） > 1^[3, 5]^作为预测PH的参考指标。CMR成像除了可通过横轴位图像测量D_PA_和D_PA_/D_Ao_外，还可应用电影序列和相位对比序列显示心动周期不同时相主肺动脉（main pulmonary artery, MPA）的形态，对各个时相的MPA径线进行测量^[6]^。

本研究旨在应用CMR比较PH患者与健康志愿者之间MPA不同时相、不同方位管径变化的差异，并探究其与RHC所测血液动力学参数间的相关性，探讨应用CMR测量MPA各径线在评估PH中的临床应用价值。

## 1 材料与方法

### 1.1 研究对象

收集2012年1月-2016年10月在天津医科大学总医院经RHC确诊为PH并接受CMR检查的患者84例。纳入标准：①首次确诊为PH且尚未经任何治疗；②RHC检查结果符合PH诊断标准；③CMR与RHC检查间隔时间小于1周；④符合CMR检查安全标准并获得患者知情同意；⑤CMR图像质量符合诊断与后处理分析要求。其中1例CMR图像质量不佳而排除，最终纳入PH组患者83例。49例健康志愿者作为正常对照组，其心率、血压均在正常范围，均无心肺疾病、代谢综合征等病史并接受了CMR检查。本研究经天津医科大学总医院伦理委员会批准，所有受检者对此项研究知情、同意。

### 1.2 CMR检查方法

采用GE 1.5T Twin-speed Infinity with Excite II超导型MR扫描仪（GE Healthcare, Milwaukee, WI, USA），8通道心脏相控阵线圈，心电门控，呼气末屏气采集。

MPA双斜位成像获取方法：首先获得横轴位非门控快速稳态进动采集序列（fast imaging employing steady-state acquisition, FIESTA）序列图像（图 1A）；在所获横轴位图像上取平行于MPA走行方向采用FIESTA序列采集MPA斜矢状位图像，显示右心室（right ventricle, RV）流出道和MPA（图 1B）；在所获斜矢状位图像中肺动脉瓣上方1.5 cm-2 cm处取垂直于血流方向定位（图 1B），采用Fast Cine相位对比（phase-contrast, PC）序列行单层多时相扫描，得到MPA横截面的幅度图（图 1C，图 1D）和相位图。FIESTA序列成像参数^[7, 8]^：TR/TE min full/min full，翻转角45°，带宽125 kHz，FOV 35 cm×35 cm，矩阵224×224，扫描层厚8 mm，无层间隔，NEX 1，扫描时相数为20。Fast Cine PC序列成像参数^[9]^：TR/TE自动选择最小重复时间/min full，翻转角20°，带宽31.25 kHz，FOV 40 cm×40 cm，矩阵256×256，NEX 1，扫描时相数为30，流速编码方向为SLICE，速度编码值为150 cm/s。

**1:**
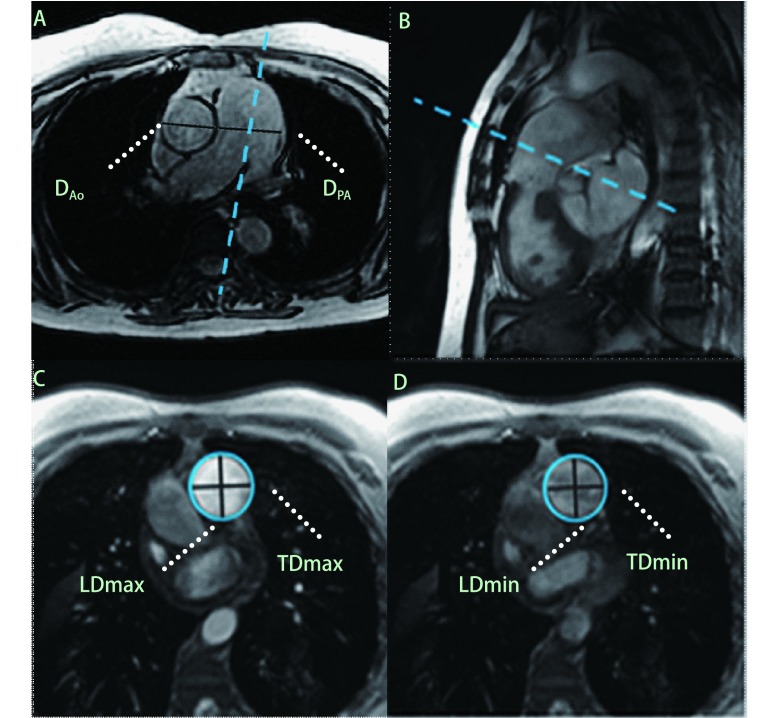
CMR、MPA图像采集及径线测量方法（A-D为同一PH患者）。A：CMR横轴位非门控FIESTA序列MPA横径最大的层面，分别测量D_PA_和D_Ao_。B：由A图取平行于MPA走行方向定位，获得RV流出道和MPA图像。C、D：由B图于肺动脉瓣上方1.5 cm-2 cm处垂直于血流方向定位，获得MPA横截面Fast Cine PC序列单层多时相图像，于幅度图上手动勾画心动周期内各时相MPA内缘，在最大、最小截面积时相分别测量MPA横径（TD）和纵径（LD）。 The measurement methods of CMR MPA diameters (A-D from the same patient). A: D_PA_ and D_Ao_ were measured from CMR non-gated FIESTA transversal image on the slice of the maximum MPA diameter. B: RV outflow tract with longitudinal MPA was acquired by localizing parallel to the direction of MPA in A. C, D: An image plane was prescribed perpendicular to the MPA flow direction and 1.5 cm-2 cm above the level of the pulmonary valve, and a single slice multi-phase cross-section images of MPA were obtained with Fast Cine PC sequence. Tracing the inner edge of MPA manually on each amplitude image during cardiac cycle was in order to measure the MPA TD and LD on the phases with the maximum and minimun area. CMR: cardiovascular magnetic resonance; MPA: main pulmonar y ar ter y; PH: pulmonary hypertension; FIESTA: fast imaging employing steady-state acquisition; D_PA_: main pulmonary artery diameter; D_Ao_: ascending aorta diameter; RV: right ventricle; PC: phase contrast; TD: transverse diameter; LD: longitudinal diameter.

### 1.3 CMR图像分析和数据测量

将CMR扫描图像传输至GE AW 4.3 MRI工作站，应用Report Card 3.7软件进行数据分析。

在CMR的横轴位非门控FIESTA图像上，选取MPA横径最大的层面分别测量MPA横径值D_PA_和升主动脉横径值D_Ao_，并计算D_PA_/D_Ao_。

在MPA横截面的PC序列幅度图上手动描点勾画心动周期各时相MPA内缘，软件自动计算各个时相MPA的截面积，在MPA最大、最小截面积的时相图上分别测量MPA横径（transverse diameter, TD）和纵径（longitudinal diameter, LD）（图 1C，图 1D），计算最大平均直径（max mean diameter, MDmax）、最小平均直径（min mean diameter, MDmin）、横径变化分数（fraction transverse diameter, fTD）、纵径变化分数（fraction longitudinal diameter, fLD）、MPA顺应性（distensibility）。计算公式如下：

MDmax=(TDmax+LDmax)/2

MDmin=(TDmin+LDmin)/2

fTD=100×[(TDmax-TDmin)/TDmin]

fLD=100×[(LDmax-LDmin)/LDmin]

Distensibility=100×[(最大截面积–最小截面积)/最小截面积]

### 1.4 血液动液力学指标的测量和体表面积的计算

对所有PH患者进行RHC检查，测量和计算MPA处的mPAP^[10]^。体表面积（body surface area, BSA）估算公式为：BSA（m^2^）=0.006, 1×身高（cm）+ 0.012, 8×体重（kg）-0.152, 9。

### 1.5 统计学方法

采用SPSS 18.0统计软件对数据进行统计。计量资料以均数±标准差（Mean±SD）表示。PH组与对照组的MPA和主动脉各相关测量结果采用多变量一般线性模型（multivariate general linear model）进行比较，以排除年龄、性别及BSA的影响。分别比较PH组及对照组的fTD和fLD。对CMR获取的MPA各参数及D_PA_/D_Ao_与RHC的mPAP进行偏相关分析，以控制协变量年龄、性别及BSA的影响。采用受试者工作特征（receiver operating characteristic, ROC）曲线计算并综合评估各指标曲线下面积（area under the curve, AUC），比较各指标评价PH的敏感度、特异度，计算约登指数（*Youden* index）以寻找评估PH状态的临界值（cut-off value）。以*P* < 0.05为差异有统计学意义。

## 2 结果

### 2.1 一般资料

PH组83例，其中女性73例，年龄[(43.7±14.2), 14-75]岁；mPAP为[(53.8±15.2), 22-94]mmHg；BSA[(1.63±0.20), 1.20-2.09]m^2^。对照组49例，其中女性39例，年龄[(37.5±10.4), 24-58]岁；BSA[(1.66±0.16), 1.39-2.12]m^2^。

### 2.2 PH组与对照组间CMR MPA各参数及D_PA_/D_Ao_比较

两组间，PH组的D_PA_、D_PA_/D_Ao_、MDmax、MDmin均明显升高（*P* < 0.001）（图 2），fTD、f LD及顺应性明显降低（*P* < 0.001）；两组间D_Ao_无明显差异（*P*=0.225）（表 1）。对照组fTD小于fLD（*P* < 0.001），而PH组两参数间无明显差异（*P*=0.3）（图 3）。

**2:**
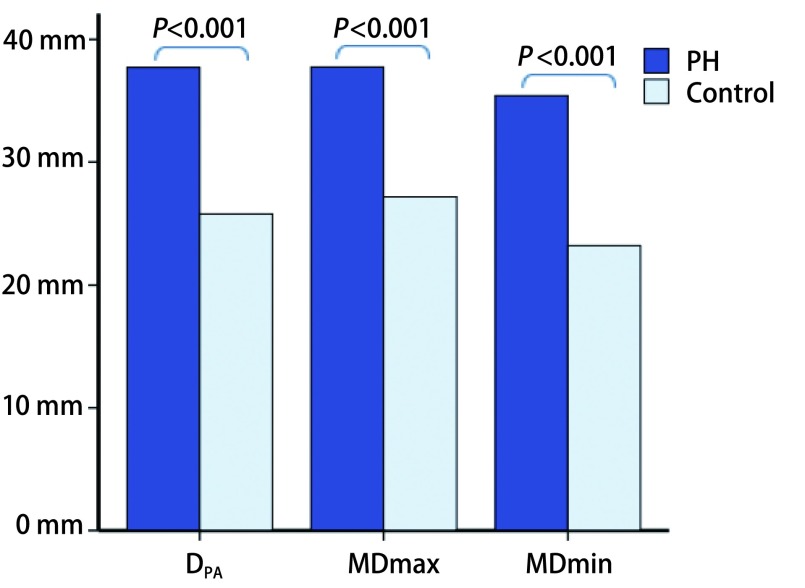
PH组与对照组间MPA径线参数比较。PH组D_PA_、MDmax、MDmin均显著升高。 Comparison of MPA vessel diameters between PH and control group. D_PA_, MDmax and MDmin were significantly increased in PH. MDmax: max mean diameter; MDmin: min mean diameter.

**1:** PH组与对照组CMR MPA、Ao各参数比较 Comparation of parameters of MPA and Ao between PH and control group

Parameters	Control group (*n*=49)	PH group (*n*=83)	*P* value
D_PA_ (mm)	25.8±3.3	37.7±5.5	< 0.001
D_Ao_ (mm)	26.8±3.2	28.3±5.0	0.225
D_PA_/D_Ao_	1.0±0.1	1.4±0.3	< 0.001
MDmax (mm)	27.1±2.5	37.7±5.5	< 0.001
MDmin (mm)	23.2±2.4	35.7±6.2	< 0.001
fTD (%)	14.1.±8.8	6.8±6.5	< 0.001
fLD (%)	21.3±11.9	7.5±6.9	< 0.001
Distensibility (%)	48.4±14.1	19.7±11.5	< 0.001
Ao: ascending aorta; D_PA_/D_Ao_: ratio of the D_PA_ and D_Ao_.			

**3:**
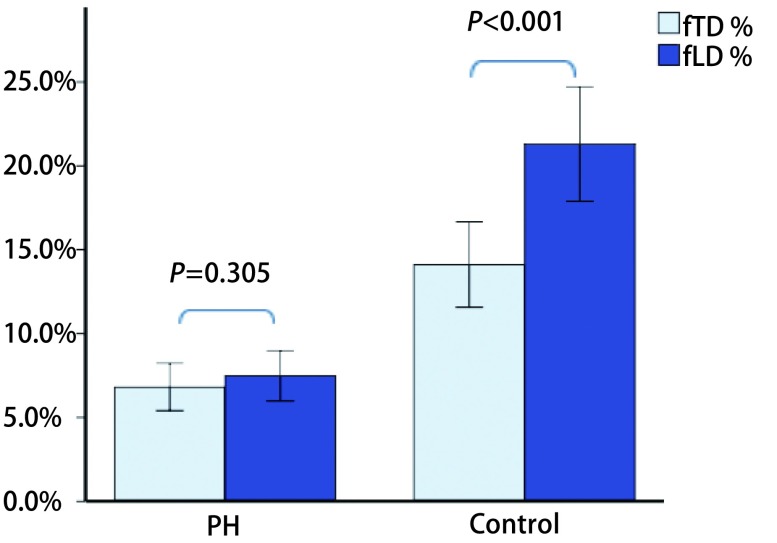
PH组及对照组各自fTD与fLD相比，对照组fTD小于fLD（*P* < 0.001），而PH组两参数间无显著性差异（*P*=0.305）。 Comparison between the fTD and fLD. FTD was smaller than fLD in control group, whereas there was no significant difference in PH. fTD: fraction transverse diameter; fLD: fraction longitudinal diameter.

### 2.3 PH组CMR MPA各参数及D_PA_/D_Ao_与mPAP间相关性

D_PA_、D_PA_/D_Ao_、MDmax、MDmin分别与mPAP呈正相关，其中D_PA_/D_Ao_相关性最强（*r*=0.534, *P* < 0.001），其次为MDmin（*r*=0.362, *P*=0.001），D_PA_与mPAP相关性较上述两参数稍弱（*r*=0.326, *P*=0.003），MDmax表现相对较弱（*r*=0.315, *P*=0.004）。顺应性、fLD、fTD分别与mPAP呈负相关，对应相关系数r值依次为-0.298（*P*=0.007）、-0.290（*P*=0.009）和-0.273（*P*=0.014）（图 4）。

**4:**
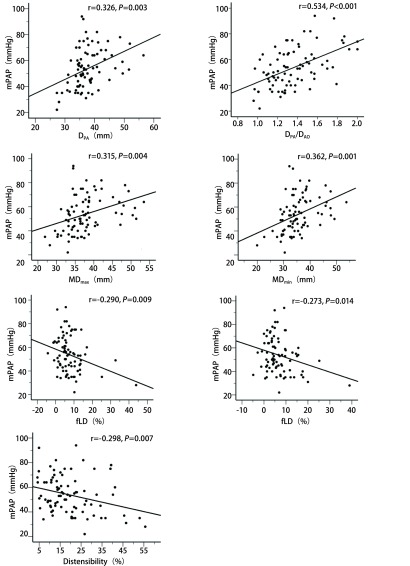
PH组MPA各参数与mPAP间的相关性的散点图。D_PA_、D_PA_/D_Ao_、MDmax、MDmin分别与mPAP呈显著正相关，其中D_PA_/D_Ao_相关性最强；顺应性、fLD、fTD分别与mPAP呈负相关。 Scatterplots showed the relationships between MPA indices and mPAP. D_PA_, D_PA_/D_Ao_, MDmax, and MDmin were positively correlated with mPAP respectively, and the strongest was found for DPA/D_Ao_ (*r*=0.534, *P* < 0.001). Disitensibility, fLD, and fTD had negative correlation with mPAP. MPA: main pulmonary artery; mPAP: mean pulmonary artery pressure.

### 2.4 CMR MPA各参数评价PH的效能

CMR MPA各参数评价PH的效能和ROC曲线分析结果见表 2、图 5。其中MDmin > 28.4 mm和MDmax > 32.4 mm的*Youden*指数（0.952, 0.904）和AUC（0.979, 0.981）最高，且两者的特异度（specificity）均达100%。D_PA_ > 30.5 mm的AUC及敏感度（sensitivity）均与MDmin > 28.4 mm相同，但其特异度稍低（0.959）。顺应性 < 26.9%、D_PA_/D_Ao_ > 1.1评价PH状态的能力相对较弱，其AUC分别为0.940、0.932。

**2:** MPA各参数预测PH的效能 Diagnostic performance of MPA parameters for predicting PH

Parameter	AUC (SE)	Cut-off value	Sensitivity	Specificity	Youden index
D_PA_ (mm)	0.979 (0.010)	30.5	0.952	0.959	0.911
D_PA_/D_Ao_	0.932 (0.022)	1.1	0.892	0.837	0.728
MDmax (mm)	0.981 (0.010)	32.4	0.904	1.000	0.904
MDmin (mm)	0.979 (0.013)	28.4	0.952	1.000	0.952
Distensibility (%)	0.940 (0.019)	26.9	0.795	0.980	0.775
AUC: area under the curve; SE: standard error.					

**5:**
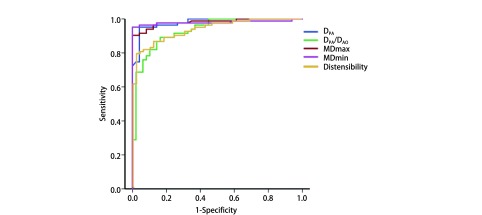
ROC曲线显示MPA各参数预测PH的效能。MDmin> 28.4 mm和MDmax>32.4 mm预测PH效能较其他参数更高（AUC=0.979, 0.981）。 ROC curve showed the ability of MPA indices to predict PH. MDmin>28.4 mm and MDmax>32.4 mm performed relative better than others in predicting PH (AUC=0.979, 0.981, respectively). ROC: Receiveroperating characteristic.

## 3 讨论

CMR作为一种成熟的无创、无电离辐射的影像检查方法，其多方位成像平面、多样化的序列优势利于精确评估血液动力学改变引起的血管径线变化。由于肺动脉的形态受其位置及走行方向的制约，使常规横轴位成像显示的MPA管径并非其真实横截面管径，因此有必要对MPA标准横截面管径与PH的关系进行评价，并探讨CMR测量MPA各径线预测PH的临床价值。

先前的研究^[11-14]^表明D_PA_和D_PA_/D_Ao_与mPAP均具有很好的相关性。在横轴位CT或非心电门控横轴位MR图像上D_PA_≥29 mm是临床最常用以预测PH的指标。Shen等^[11]^对2014年以前的基于CT测量MPA径线的文献进行系统回顾和分析，发现各研究报道的D_PA_检测PH的临界值为25 mm-33.2 mm。本研究结果的D_PA_临界值为30.5 mm，也在该范围内。然而横轴位图像显示的并非是MPA的标准横截面管径，而且非心电门控扫描获得的图像时相不定，心脏大血管搏动伪影也可造成MPA管壁模糊，这些因素可能是造成各研究所得检测阈值不同的重要因素^[4, 11]^。研究结果表明，D_PA_/D_Ao_与mPAP的相关性（*r*=0.534, *P* < 0.001）较D_PA_（*r*=0.326, *P*=0.003）强，预测PH的临界值为1.1。早期的MR研究也有类似发现，D_PA_/D_Ao_预测mPAP（mPA*P*=3.7+24×D_PA_/D_Ao_; *r*=0.7, *P* < 0.01）比D_PA_强^[12]^。最近Karakus等^[13]^的研究也得出D_PA_/D_Ao_与MPA各径线或其他RV形态学参数相比，与mPAP相关性更好、重复性高，且为不良事件的独立危险因素^[5, 13]^。人体自身特征因素如体型等，可同时影响D_PA_和D_Ao_，因此D_PA_/D_Ao_能在一定程度上有效地校正混杂因素^[15]^。

本研究发现MDmin > 28.4 mm和MDmax > 32.4 mm预测PH的效能最高，MDmax、MDmin分别反映了收缩期、舒张期MPA真实横截面的平均直径，由于其横截面并非标准圆形，因此计算平均直径能有效减小MPA径线测量偏差。Burman等^[6]^通过对正常人MPA最大、最小平均直径与年龄、BSA和性别的回归模型分析，发现舒张期平均直径测量值与上述因素之间的相关性比收缩期更稳定（*R*^2^值更高），提示在多因素分析中MDmin可能对评估肺动脉病理性扩张更具潜在优势。

对照组MPA横截面的横、纵径变化幅度（fTD和fLD）并非一致，fTD明显小于fLD（*P* < 0.001），这提示正常人MPA管径纵向变化程度较横向稍高，其原因可能是MPA横向紧邻升主动脉，而纵向仅有心包纤维组织的束缚且心包弹性较大。PH组的fTD、fLD均较正常人群显著降低（*P* < 0.001），且两者间的差异不明显（*P*=0.305），可能提示了血液动力学状态发生改变导致PA的重构，纵向代偿能力降低更明显，但这需要进一步的组织学及病理生理学证实。PH患者的fTD和fLD均与mPAP呈负相关，其机制可能与MPA顺应性降低有关。顺应性可反映血管壁的搏动度，其降低与mPAP升高相关，PA管壁重构以适应肺血管压力升高，使得管壁硬度增加，进而PA搏动幅度下降、扩张受限^[16, 17]^。虽然fTD、fLD和顺应性均与mPAP相关，但前两者与mPAP的相关系数较顺应性低（图 4），可能由于血管面积比单一径线更能反映整个血管的改变。本研究显示顺应性 < 26.9%预测PH的敏感度为79.5%、特异度为98%。Creuze等^[18]^研究得出的顺应性阈值为≤20%，阈值的差异可能与样本差异有关。

综上，常规横轴位CMR图像D_PA_ > 30. 5 mm、D_PA_/D_Ao_ > 1.1可作为提示PH的参考标准，且方法简便；MPA标准横截面心电门控PC图像MDmin > 28.4 mm、MDmax > 32.4 mm预测PH效能很高。CMR无创性测量MPA径线对于筛查PH、评估其严重程度、治疗后随访等，均具有临床实用价值。
